# Tailoring topology and bio-interactions of triazine frameworks

**DOI:** 10.1038/s41598-024-64787-x

**Published:** 2024-06-26

**Authors:** Sara Bagheri, Mohsen Adeli, Abedin Zabardasti, Siamak Beyranvand

**Affiliations:** 1 Faculty of Science, Department of Chemistry, Lorestan University, Khorramabad, Iran; 2 Department of Biology, Chemistry, Pharmacy Institute of Chemistry and Biochemistry, Freie Universität Berlin, Berlin, Germany

**Keywords:** Triazine frameworks, Optical activity, Mechanochemistry, Antibacterial activity, Enantiorecognition, Materials chemistry, Organic chemistry, Polymer chemistry

## Abstract

The construction of covalent organic frameworks with special geometery and optical properties is of high interest, due to their unique physicochemical and biological properties. In this work, we report on a new method for the construction of triazine frameworks with defined topologies using coordination chemistry. Ball milling and wet chemical reactions between cyanuric chloride and melamine were directed in spatial arrangements and opposite optical activity. Cobalt was used as a directing agent to drive reactions into special morphologies, optical properties and biological activity. The enantiorecognition ability of triazine frameworks that was manifested in their activities against bacteria, demonstrated a new way for the construction of materials with specific interactions at biointerfaces.

## Introduction

Synthesis of chiral organic frameworks with a specific 3D structure has attracted a great deal of attention in the past several years^1,2^. Spatial arrangement at atomic level manifests not only in the morphology and optical activity of materials but also their interactions at biointerfaces^3^. Chirality is the basic factor determining the biological properties and function of many biopolymers including polypeptides, proteins, nucleic acids and their monomers^4–6^. Synthesis of biopolymers with optical activity inside body is catalyzed by enzymes and take place at ambient conditions efficiently^7^. However, their synthetic mimics are constructed via multi-step chemical reactions which are time-consuming and sometimes non-reproducible^8,9^. Construction of optically active biopolymers with the specific interactions at biointerfaces is an efficient strategy to create new vectors with desired biological functions^10^. A straightforward method for the induction chirality in nanomaterials and polymers is to attach chiral elements to their functional groups or assemble them noncovalently^11,12^. This approach give rise to the same optical activity as the conjugated chiral element and do not induce intrinsic chirality^13^. In the other words, in this approach polymers and nanomaterials are platforms carrying chiral molecules rather than being chiral scaffolds^14,15^. On the other side, the density of chiral molecules and their positions and arrangements are not well controlled in this method, leading to less-defined materials with low reproducibility^16^. In recent years, different parameters including chiral solvents, chiral molds, and circular polarized light have been used to induce chirality in macromolecules^17–20^. These factors influence the spatial arrangements and assembly of polymers that in turn result in an intrinsic chirality^21^. Taking advantage of this method covalent organic frameworks with specific optical activities and enantiorecognition have been synthesized^22^. Recently, chiral covalent organic frameworks have been synthesized from achiral precursors in the presence of catalytic amount of a chiral molecule^22^. The chiral frameworks have shown enantioselectivity against chiral carbohydrates and been used for the asymmetric Henry reaction, after modification with copper ions. This is a high achievement toward construction of covalent organic frameworks in which immobilization of monomers is derived by a chiral molecule at the molecular level. This method is limited by catalytic chiral molecules and cannot be performed in a complete achiral medium.

In this work, we report on a method for the construction of optically active covalent organic frameworks using cheap precursors without needing complex catalysts. Ball milling and wet chemical reactions between cyanuric chloride and melamine were directed into special directions to form optically active triazine frameworks (OTFs) with high enantioselectivity toward histidine. This enantioselectivity was manifested in their activities against bacteria, indicating high potential of this method for the manipulation of interactions of COFs at biointerfaces.

## Results

**Figure 1:**
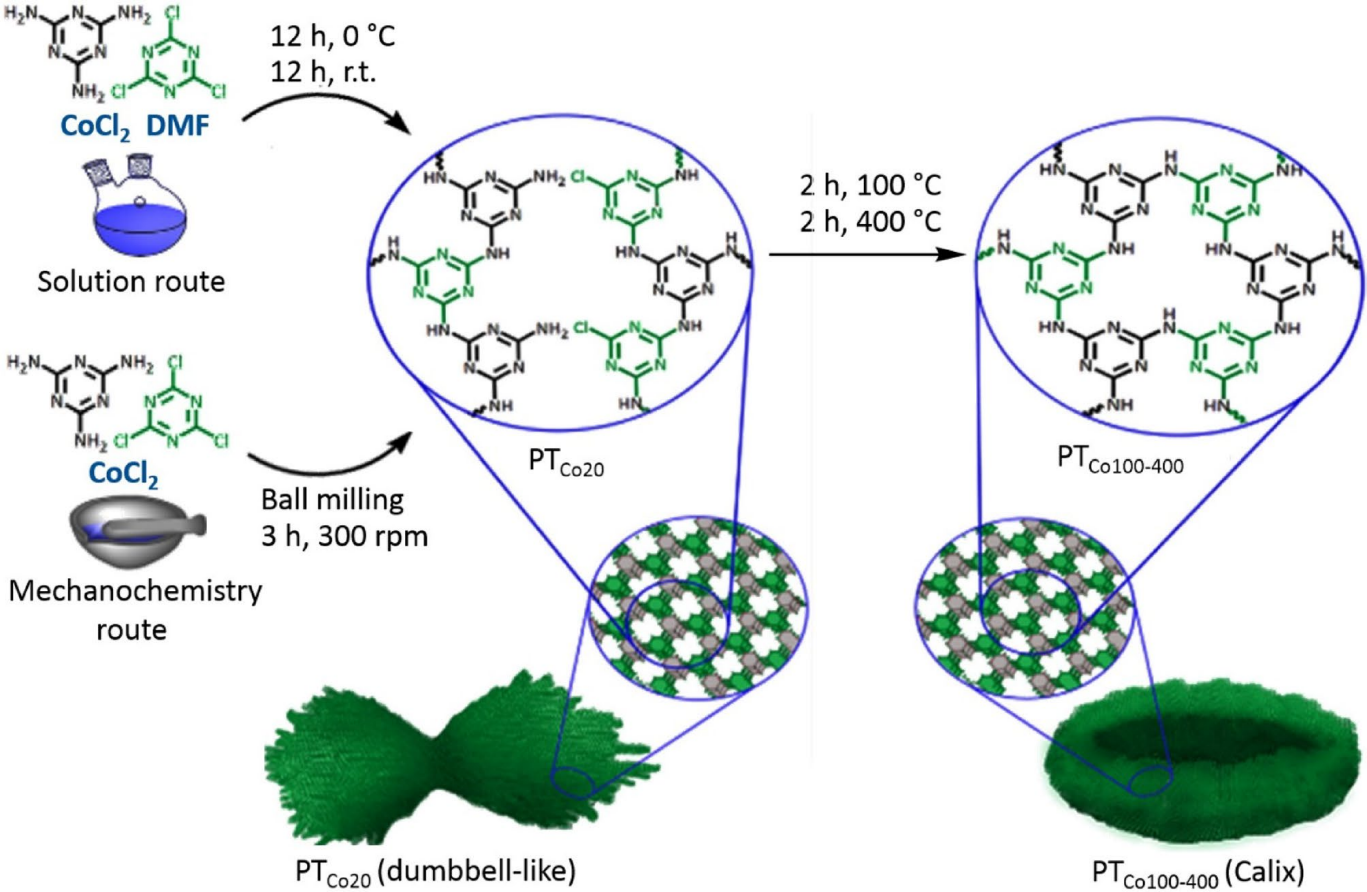
Schematic representation of the synthesis of triazine covalent organic frameworks by two methods including solution and mechanochemistry. Coordination chemistry mediated by cobalt ions was the main driving force to obtain chiral frameworks with special morphologies.

Induction of special geometry in covalent organic frameworks (COFs) and tunning their morphology plays a vital role in their future applications. In this work, triazine covalent organic frameworks were constructed by nucleophilic reaction between melamine and cyanuric chloride at different conditions. In order to control the morphology of triazine covalent organic frameworks, reactions were performed in the presence of cobalt ions as directing agent. Moreover, reactions were performed in solution and dry states to investigate effect of solvent on the optical property and morphology of the obtained frameworks (Fig. 1). Triazine and melamine were mixed either in solvent or dry state (ball milling) in the presence of cobalt ions at 20 °C. The product of this reaction was then heated up to 400 °C and collected for further investigations in terms of composition, crystallinity and morphology. This protocol is milder than ionothermal method and cobalt ions can be easily removed after reaction, due to their weak interactions with framework. In the conventional ionothermal method, monomers are dissolved in molten zinc chloride and heated up to 400 °C^23^. Carbonization at high temperatures is one of the main challenging issues in ionothermal method^24^.

Covalent organic frameworks synthesized by wet chemical reaction and mechanochemical method abbreviated as PTxy and MPTxy, respectively, where PT refers to polytriazine and the symbols x and y correspond to the metal cation and temperature of reaction.

**Figure 2:**
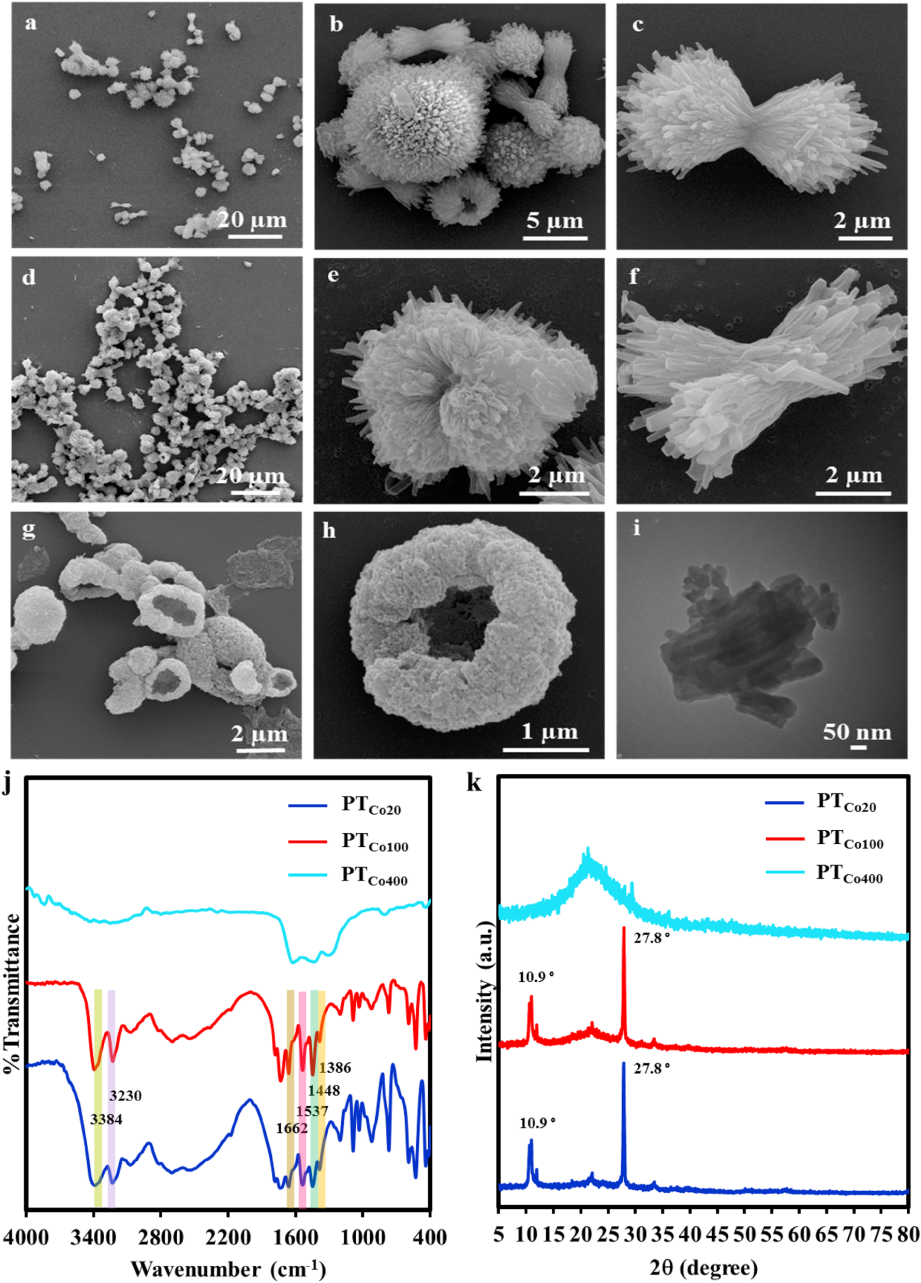
SEM images of the synthesized frameworks in solution. (**a**–**c**) PT_Co20_ and (**d**–**f**) PT_Co100_ showed dumbbell-like morphology. SEM image of (**g**, **h**) PT_Co400_ with a calix morphology, confirming deformation of frameworks at high temperatures. (**i**) TEM image of PT_Co100_ displaying half of a dumbbell-like object which is formed by assembly of rod-like objects. (**j**) IR spectra and (**k**) XRD diffractograms of frameworks synthesized in solution.

The morphology of COFs was investigated using scanning electron microscopy (SEM) and transmission electron microscopy (TEM). The organization and orientation^25^ of monomers by cobalt ions led to COFs with dumbbell-like morphology at 20 °C and 100 °C (Fig. 2a–f and S2). In order to prove the role of cobalt ions in directing frameworks to such a morphology, a control reaction was performed in the absence of metal ions (Fig. S1). Any special morphology for the product was not observed, indicating their crucial role to render frameworks dumbbell objects. Heating COFs to 400 °C, however, changed them to irregular objects (Fig. 2g, h). Based on TEM images, dumbbell-like COFs were made of rod-like objects with 107 nm and 496 nm in diameter and length respectively (Fig. 2i).

Vibrational absorbance bands at 3384 cm^−1^ and 3230 cm^−1^ in the IR spectra of PT_Co20_ and PT_Co100_ were assigned to their amino functional groups. They showed 83–187 cm^−1^ chemical shift in comparison with melamine, indicating their interactions with cyanuric chloride by hydrogen bonding and changing to secondary amino functional groups upon replacement of chlorine atoms (Fig. 2j). The absorbance bands at 1662 cm^−1^, 1537 cm^−1^ and 1448 cm^−1^ were assigned to the bending vibration of N–H and stretching vibrations of C=N and C–N bonds, respectively. The absorbance band at 1386 cm^−1^, assigned to the stretching vibration of C-NH-C bonds, was a further prove for melamine-cyanuric chloride connections^26^. These absorbance bands indicated that PT_Co20_ and PT_Co100_ are composed of triazine rings crosslinked by secondary amino functional groups. The data of IR spectrum in PT_Co400_ indicate the change of triazine units due to heating Disappearing absorbance bands of amino functional groups and broadening absorbance bands of C=N and C–N bonds in the IR spectra of PT_Co400_ confirmed carbonization and partial decomposition of this compound at high temperatures.

The C/N ratio for PT_Co20_ and PT_Co100_ was 0.5, close to the calculated value (0.6), indicating the expected composition and chemical formula shown in Fig. 1. The low cobalt content (~ 0.1 wt%) of PT_Co20_ and PT_Co100_ was counted for the weak interactions between monomers and cobalt ions and their exclusion upon workup and purification (Table S1). However, the C/N ratio of PT_Co400_ was higher than the calculated value, due to the carbonization of this compound at higher temperatures which is in agreement with the SEM image shown in Fig. 2g and h (Table S1).

**Figure 3:**
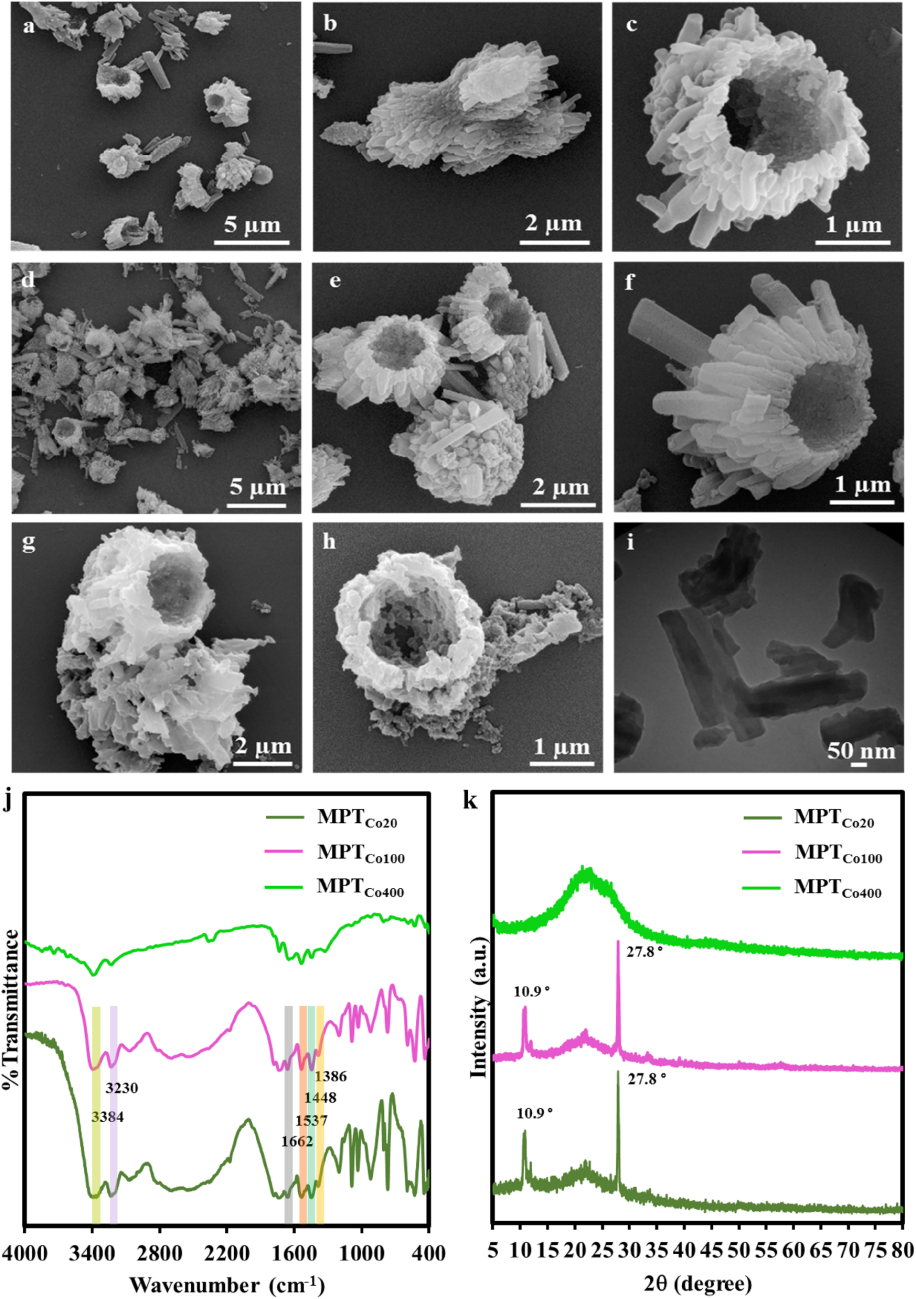
SEM images of frameworks synthesized in dry state by ball milling. (**a**–**c**) SEM images of MPT_Co20_ showed calix morphology for this compound. (**d**–**f**) SEM images of MPT_Co100_ indicated calix structures with a 1.2 μm cavity. (**g**, **h**) SEM image of MPT_Co400_. Such calix objects were hardly found for this sample. (**i**) TEM image MPT_Co100_ indicating rod-like building blocks for this compound. (**j**) IR spectra and (**k**) XRD diffractograms of frameworks synthesized by ball milling.

In order to investigate the effect of solvent on the morphology of triazine covalent frameworks, they were synthesized in dry state using ball milling. Monomers were mixed with CoCl_2_ and ball milled at 20 °C and 300 rpm for 3 h and then product was heated to 100 °C and 400 °C. Frameworks constructed at 20 °C and 100 °C showed a calix morphology (Figs. 3c,e). A closer look revealed that calix objects are consisting assembled rod-like structures (Fig. 3a, b, d, f and Fig. S4). To gain more information about the structure of calix frameworks, they were sonicated at room temperature for 30 s and their TEM images were recorded.

Rod-like structures with 97 nm in diameter and 465 nm length were detected as the building blocks of calix frameworks. Disassembly of rod-like structures by gentle and short-time sonication indicated that they are associated by noncovalent interactions to make calix frameworks. This is similar to assembly of different domains of proteins in a three-dimensional structure with special function (Fig. 3i). The morphology of MPT_Co100_ was changed to irregular shapes after heating up to 400 °C, indicating noncovalent interactions between rod-like building blocks and their dissociation at high temperatures (Fig. 3g, h).

Absorbance bands at 3384 cm^−1^ and 3230 cm^−1^ in the IR spectra of MPT_Co20_ MPT_Co100_ were corresponding to primary and secondary amino functional groups. Reaction between melamine and cyanuric chloride manifested in 83–187 cm^−1^ shift of absorbance bands of amino functional groups to lower frequencies in comparison with melamine. The absorbance bands at 1537 cm^−1^, 1448 cm^−1^ and 1386 cm^−1^ were corresponding to C=N, C–N and C-NH-C bonds, respectively, indicated that MPT_Co20_ and MPT_Co100_ are composed of triazine rings connected by secondary amino groups (Fig. 3j). The IR spectra of MPT_Co400_ was different from MPT_Co20_ MPT_Co100_, indicating dramatic changes in the structure of frameworks at high temperature. The absorbance bands related to the primary and secondary amino functional groups are disappeared and absorbance bands of the aromatic rings are broadened. All these changes indicated carbonization of framework at high temperature.

The C/N ratios for MPT_Co20_ and MPT_Co100_ obtained by elemental analysis was the same as the calculated value (~ 0.6) and fitted well with the chemical formula shown in Fig. 1. The cobalt content of MPT_Co20_, MPT_Co100_ and MPT_Co400_ was low, confirming the successful exclusion of metal ions by workup and purification (Table S2).

Two distinct and sharp diffraction peaks in the powder X-ray diffractograms of PT_Co20_ and PT_Co100_ were corresponding to long-range order in their structures. Peak (100) at 2θ 10.9° was attributed to definite d-spacing of 0.8 nm in triazine frameworks^27–29^. The stronger peak (002) at 2θ 27.8° was assigned to interlayer distance about 0.3 nm^30–34^. X-ray diffractograms of precursors are shown in Fig. S8. As it can be seen, peaks of starting materials are not appeared in the XRD diffractograms of triazine frameworks, indicating successful synthesis and purification of this materials. XRD diffractograms for frameworks synthesized in solution and by ball milling were very similar and showed the same crystallinity, indicating no significant affect from solvent on the primary structure of triazine frameworks. Sharp peaks at 10.9 2θ and 27.8 2θ were omitted by heating frameworks up to 400 °C and a broad peak at 15–30 2θ was appeared, confirming decomposition of their structure at high temperatures.

This is confirmed by SEM images of triazine framework after heating up to 400 °C, where the morphology of triazine frameworks is changed, indicating disruption of the morphology and network of the triazine framework (Figs. 2k, 3k).

**Figure 4:**
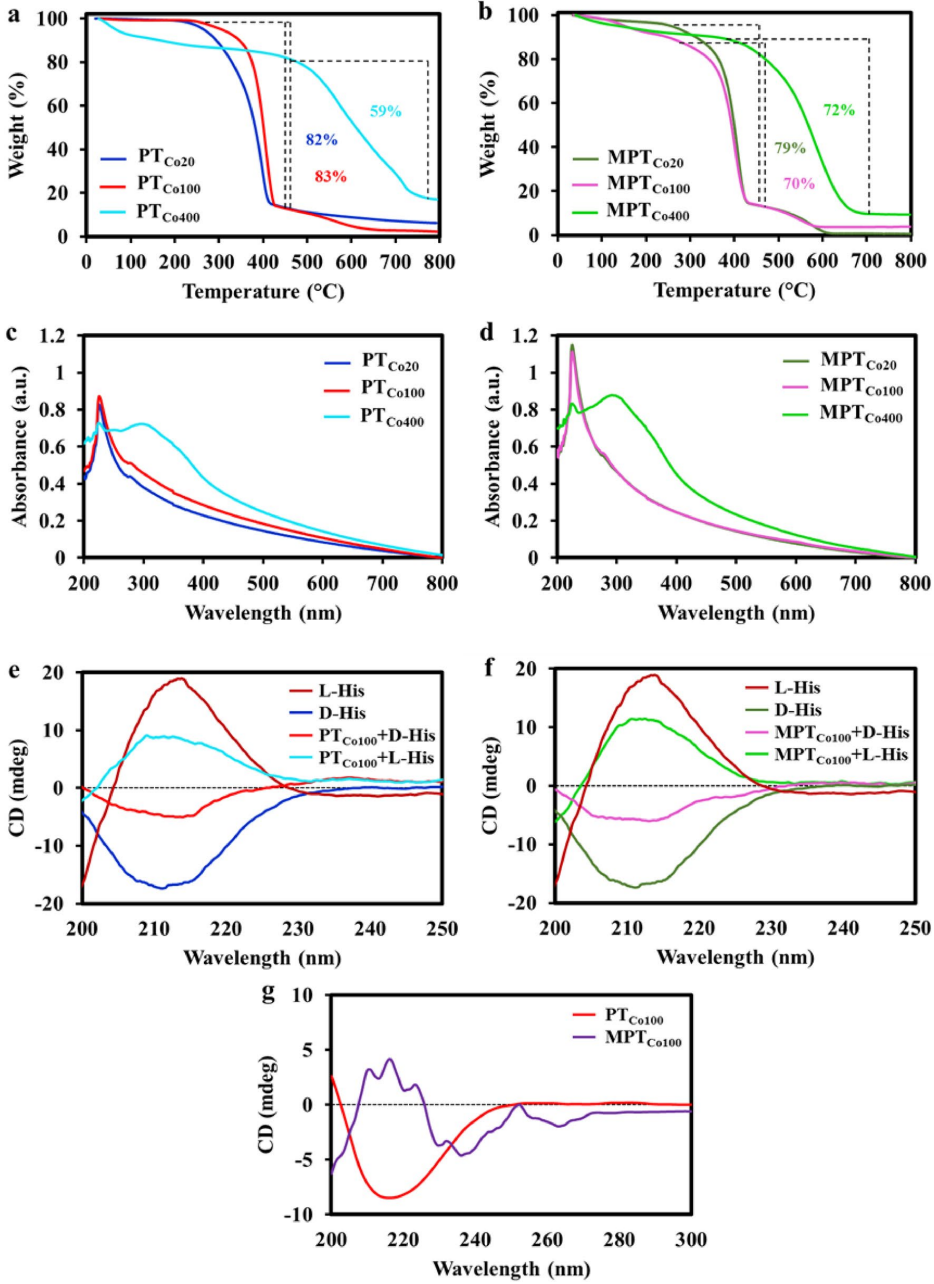
TGA thermograms of (**a**) PT_Co20_, PT_Co100_, PT_Co400_ and (**b**) MPT_Co20_, MPT_Co100_, MPT_Co400_ recorded at 25 °C-800 °C temperature range under argon. (**c**, **d**) UV spectra of triazine covalent organic frameworks synthesized in solution and by ball milling. (**e**, **f**) CD spectra of stock solutions of L- and D-histidine and supernatant of their solution after incubation with PT_Co100_ and MPT_Co100_ for 48 h at room temperature. (**g**) CD spectra of PT_Co100_ and MPT_Co100_ in ethanol solvent after 48 h shaking at room temperature.

The thermal stability of triazine covalent organic frameworks were evaluated using thermogravimetric analysis (TGA). Thermograms of PT_Co20_ and PT_Co100_ as well as MPT_Co20_ and MPT_Co100_ were very similar with a main weight loss at 300 °C–430 °C. This weight loss was due to detachment of nitrogen atoms and carbonization of triazine frameworks. The thermal behaviour of the synthesized frameworks were similar to those reported for in literature^35,36^ (Fig. 4a, b). Higher stability of PT_Co400_ and MPT_Co400_ in comparison to other triazine frameworks indicated carbonization of these frameworks at higher temperatures and forming platforms similar to graphite.

The optical properties of triazine covalent organic frameworks were investigated by UVs and circular dichroism (CD) spectroscopy. The UV spectra of PT_Co20_, PT_Co100_, MPT_Co20_ and MPT_Co100_ were similar with a broad absorption from 200 to 800 nm. This broad absorption with a λmax in short wavelengths and a tail up to 750 nm has been abundantly observed for frameworks with large π conjugated systems. While absorption peak at 228 nm and 275 nm were corresponding to π-π* and n-π* transitions, the shoulder at higher wavelengths was due to the extended π conjugated system^37,38^ (Fig. 4c, d). After heating samples up to 400 °C, their absorption spectra showed a new λmax at 300 nm. This red-shift was assigned to carbonization of frameworks toward a π conjugated carbon scaffold^39^.

While physicochemical and thermal properties as well as morphology of triazine covalent organic frameworks synthesized by ball milling and in solution were the same, they showed opposite optical activities. PT_Co100_ and MPT_Co100_ with defined morphologies and crystallinity were used for further investigations and their circular dichroism (CD) spectra were recorded in the range of 200–250 nm. Surprisingly, PT_Co100_ and MPT_Co100_ showed negative and positive peaks at the same areas, indicating higher structures for these frameworks with the opposite orientations (Fig. 4g).

## Discussion

A possible mechanism for the action of metal can be explained by chiral pockets. A chiral pocket can be formed upon interactions between monomers and metal ions. Coordination of bulky monomers around the metal centre results in pockets with special dimensions, due to the coordination geometry of metal. The produced chiral pocket restricts the movements of molecules around the coordinated substrate and directs the reaction in special geometries and chirality. To obtain a specific product, the accommodation of the monomers into the chiral pocket and the efficient shielding of other active sites should drive reaction in certain directions^40–42^.

We were persuaded by the opposite optical activities of PT_Co100_ and MPT_Co100_ to follow their abilities for the enantiorecognition of optically active molecules. D- and L-Histidine were incubated with the synthesized frameworks and their interactions were investigated using CD spectroscopy.

The affinity of both PT_Co100_ and MPT_Co100_ to uptake D-histidine was more that L-histidine indicating a good enentioselectivity for both compounds (Fig. 4e, f). Our calculations showed higher enentioselectivity for MPT_Co100_ than PT_Co100_.Interactions between 3D structures and living systems are governed by spatial arrangements and chirality of their components. Moreover, antibacterial activity of organic and biogenic frameworks follow different mechanisms including physical interactions and chemical pathways^43–50^. For example, frameworks with atomic catalytic centers are able to incapacitate bacteria via chemical approachs^51^. Also, composites of covalent organic frameworks with cellulose is able to antibacterial and antifouling properties by exposing to visible light^52^.

**Figure 5:**
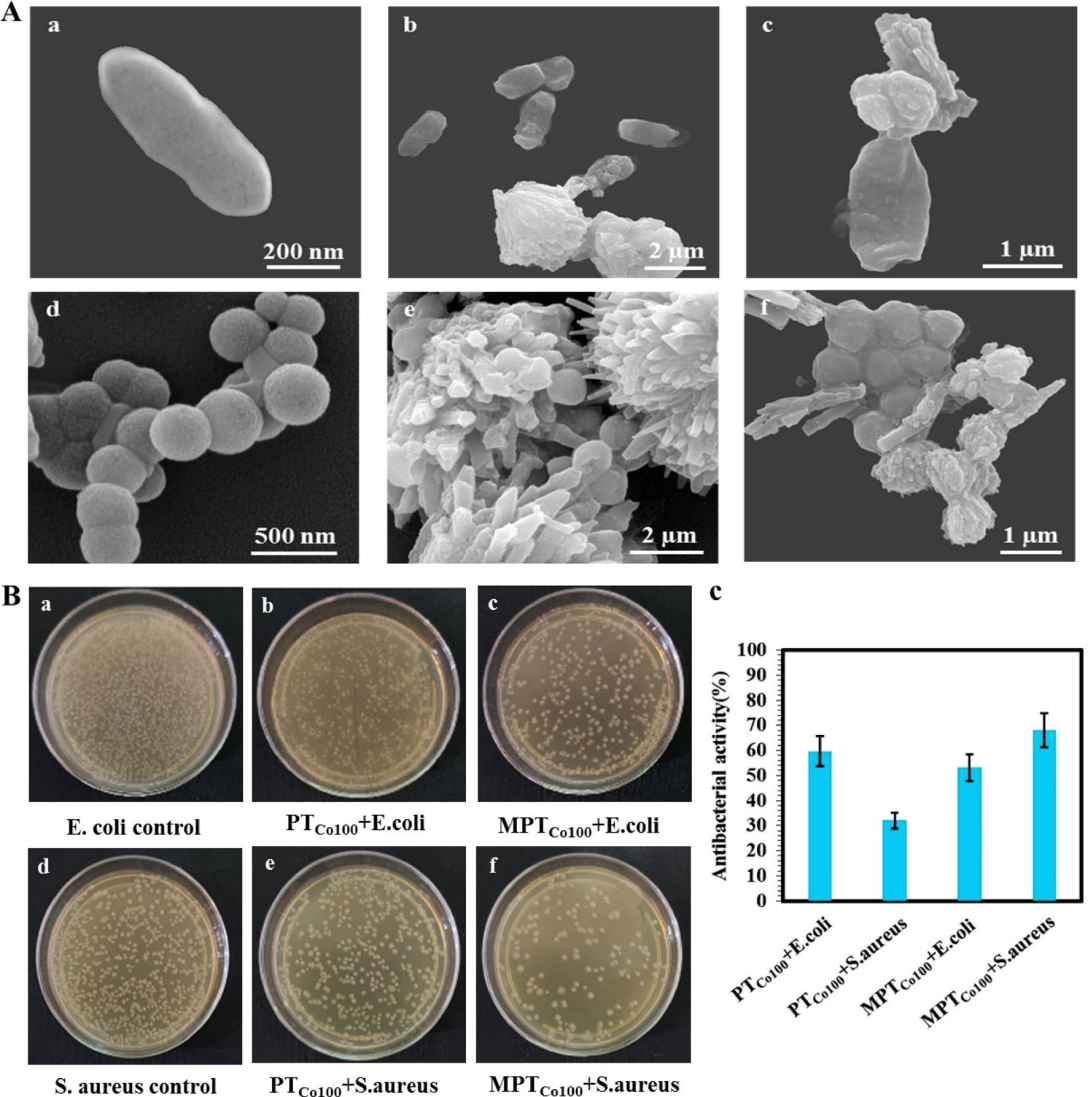
(**A**) SEM images of E. coli before (a) and after incubation with PT_Co100_ (b) and MPT_Co100_ (c). SEM image of S. aureus before (d) and after incubation with PT_Co100_ (e) and MPT_Co100_ (f). (**B**) Photographs of colonies of: E. coli before (a) and after incubation with PT_Co100_ (b) and MPT_Co100_ (c). S. aureus before (d) and after incubation with PT_Co100_ (e) and MPT_Co100_ (f). (**C**) Antibacterial activity of PT_Co100_ and MPT_Co100_ versus E. coli and S. aureus.

Regardless of the type of interactions between our synthesized frameworks and bacteria, any difference between their antibacterial activity is due to difference in their chirality. Because, their composition is almost the same. In order to evaluate the role of enantioselectivity of PT_Co100_ and MPT_Co100_ in biointeractions, their antibacterial activities against gram positive and gram-negative bacteria were investigated. Colony counting showed a clear difference between the control and bacteria incubated with the synthesized materials (Fig. 5B and C). Based on SEM images both frameworks were able to interact with E. coli bacteria. The morphology of bacteria changed upon interactions with both PT_Co100_ and MPT_Co100_ and cytoplasm of bacteria was leaking out leaving an empty and collapsed membrane (Fig. 5A and Fig. S9).

However, antibacterial activity of MPT_Co100_ against S. aureus was two times more than that for PT_Co100_. Moreover, antibacterial activity of PT_Co100_ against E. coli was almost two times than its activity against S. aureus. Difference between antibacterial activity of MPT_Co100_ and PT_Co100_ against gram-positive and gram-negative bacteria was assigned to their enantioselectivity and spatial arrangements, because all other physicochemical properties were almost the same.

The porosity of PT_Co100_ and MPT_Co100_ was also evaluated by N_2_ adsorption isotherm (Fig. S11). Similar adsorption behaviors, indicating a type II isotherm, for both structures were obtained^53^. The surface area of PT_Co100_ and MPT_Co100_ was estimated to be 19.92 and 14.37 square m^2^/g, respectively. The average pore size of the structures is 4.5 nm, which shows the mesoporous characteristics of the structures^54^ (Figure b and d of S11). In addition, the average pore diameters of PT_Co100_ and MPT_Co100_ were measured as 11.26 and 10.20 nm, respectively.

## Conclusions

Metal directed polymerization of monomers bearing heteroatoms is an efficient strategy for the construction of organic frameworks with desired topology and chirality. Metal ions coordinate and organize monomers in special geometries and drive polymerization in defined morphologies and higher structures. The chirality of the obtained frameworks is not only manifested in their enantiorecognition but also interactions at biointerfaces. This method can be used to specify interactions between frameworks and different biomolecules in living systems.

## Methods

### Synthesis of CTFs using wet chemical reactions and mechanochemistry

Polytriazine frameworks were synthesized by wet chemical reaction and abbreviated as PTxy, where PT refers to polytriazine and the symbols x and y correspond to the metal cation and the temperature at which the reaction was carried out, respectively. Polytriazine frameworks obtained by mechanochemical method were abbreviated as MPTxy, where M refers to chemical mechanochemical route.

### Synthesis of PT_Co20_

A mixture of melamine (300 mg, 2.37 mmol), cyanuric chloride (438 mg, 2.37 mmol) and anhydrous cobalt chloride (926 mg, 7.13 mmol) were added to a round bottom flask containing 45 ml DMF. Then the blue suspension was stirred at 0 °C and room temperature, each 12 h, under nitrogen. The obtained precipitate was filtered and washed with distilled water, DMF, dichloromethane and acetone. The product was dried at 70 °C. A white powder (0.34 g, yield, 20%) was obtained.

### Synthesis of MPT_Co20_

Melamine (300 mg, 2.37 mmol) cyanuric chloride (438 mg, 2.37 mmol) and anhydrous cobalt chloride (926 mg, 7.13 mmol) was added to a 50 ml stainless steel grinding bowl with six grinding balls 10 mm in diameter. This mixture was milled at room temperature and 300 rpm for 3 h with 15 min rotation and 5 min rest intervals. The product was washed by distilled water, DMF, dichloromethane and acetone and dried at 70 °C. A white powder (0.3 g, yield, 18%) was obtained.

### Synthesis of PT_Co100_ and MPT_Co100_

PT_Co20_ (165 mg) or MPT_Co20_ (165 mg) were added to a tube furnace. The furnace was heated from room temperature to 100 °C with 33 °C/min heating rate and left at the same temperature for 2 h. Afterwards, they were cooled down to room temperature with 33 °C/min cooling rate and the product was collected. White powder with yield 96% was obtained.

### Synthesis of PT_Co400_ and MPT_Co400_

PT_Co20_ (250 mg) or MPT_Co20_ (250 mg) were added to a tube furnace. The furnace was heated up to 400 °C with a 33 °C/min heating rate and left at the same temperature for 2 h. Then, it was cooled down to room temperature with 33 °C/min cooling rate and product was collected. Brown powder with yield 1.6% was obtained.

### Control reaction to study the directing role of metal ions

To investigate the role of metal ions as directing agents, a reaction between cyanuric chloride and melamine in DMF solvent in the absence of metal ions was performed. This reaction was carried out for 12 h at 0 °C and 12 h at room temperature under nitrogen atmosphere.

### CD spectra of PT_Co100_ and MPT_Co100_

To record CD spectra, 1 mg of PT_Co100_ and MPT_Co100_ compounds was dispersed in 10 ml ethanol and shaken for 48 h. Then CD spectra of dispersion was recorded at room temperature.

### Enantioselectivity of PT_Co100_ and MPT_Co100_

Solutions of L and D histidine in distilled water (500 ppm) and aqueous dispersions of PT_Co100_ and MPT_Co100_ (100 ppm) were prepared. Then, 3600 µl of histidine solutions were added to 5 ml of aqueous dispersion of PT_Co100_ and MPT_Co100_ and after 48 h shaking, the dispersion was centrifuged and the supernatant was collected for CD analysis.

### Antibacterial test

The antibacterial activity of PT_Co100_ and MPT_Co100_ against Gram-negative (E. coli) and Gram-positive (S. aureus) bacteria was investigated by colony counting method. Bacteria of Escherichia coli (E. coli) and Staphylococcus aureus (S. aureus) were cultured on nutrient agar plates at 37 °C for 24 h. A suspension of bacteria equivalent to the turbidity of 1.5 × 10^8^ CFU/ml was prepared. Then 200 μl of the suspension was added to 500 μg/ml of PT_Co100_ and MPT_Co100_ samples. In the next step, the suspension containing the bacteria and the sample was incubated for 24 h on the agar plate at 37 °C. Finally, the colonies were counted and test was repeated three times.

### SEM images of *bacteria*

To investigate the antibacterial activity of PT_Co100_ and MPT_Co100_ against S. aureus and E. coli, as well as evaluating the morphology of the bacteria before and after interaction with these materials their SEM images were recorded. For this purpose, the suspension containing bacterial solution (before and after interaction with PT_Co100_ and MPT_Co100_) was washed three times with PBS solution and immersed in 2.5% glutaraldehyde solution for 2 h at 4 °C. Then they were dehydrated with 50, 70, 85, 90 and 100% ethanol. The sample was placed on a silica substrate and by sprinkling a gold layer on it, their SEM images were recorded.

### Supplementary Information

Supplementary Information.

## Data Availability

The datasets used and/or analysed during the current study available from the corresponding author on reasonable request.
