# Author Correction: Tailoring topology and bio-interactions of triazine frameworks

**DOI:** 10.1038/s41598-024-69801-w

**Published:** 2024-08-13

**Authors:** Sara Bagheri, Mohsen Adeli, Abedin Zabardasti, Siamak Beyranvand

**Affiliations:** 1 Faculty of Science, Department of Chemistry, Lorestan University, Khorramabad, Iran; 2 Department of Biology, Chemistry, Pharmacy Institute of Chemistry and Biochemistry, Freie Universität Berlin, Berlin, Germany

Correction to: *Scientific Reports* 10.1038/s41598-024-64787-x, published online 26 June 2024

The original version of this Article omitted an affiliation for Mohsen Adeli and Siamak Beyranvand. Their correct affiliations are listed below.

Faculty of Science, Department of Chemistry, Lorestan University, Khorramabad, Iran.

Department of Biology, Chemistry, Pharmacy Institute of Chemistry and Biochemistry, Freie Universität Berlin, Berlin, Germany.

The original Article has been corrected.

