# Supplementary material for: Tailoring topology and bio-interactions of triazine frameworks
Source: Sci Rep. 2024 Jun 26;14:14777. doi: 10.1038/s41598-024-64787-x (PMC11208503; doi:10.1038/s41598-024-64787-x)
Supplement: Supplementary file 1 — Supplementary Information. [file 41598_2024_64787_MOESM1_ESM.docx]

Tailoring topology and bio-interactions of triazine frameworks

Sara Bagheri ^a^, Mohsen Adeli *^a^, Abedin Zabardasti ^a^ and Siamak Beyranvand ^a^

^a^ Faculty of Science, Department of Chemistry, Lorestan University, Khorramabad, Iran

Materials

Anhydrous cobalt chloride (CoCl_2_) and 2,4,6-trichloro-1,3,5-triazine (cyanuric chloride) was purchased from Sigma-Aldrich. 2,4,6-triamino-1,3,5-triazine (Melamine), dimethylformamide (DMF), dichloromethane and acetone were purchased from Merck (Germany). D-histidine and L-histidine were purchased from Merck company. Standard strains E. coli (ATCC 10596) and S. aureus (ATCC 25925) was prepared from Scientific and Industrial Research Organization.

Characterization

IR spectra were recorded using Shimadzo 8400 IR spectrometer by KBr tablets in the desired range. UV-Visible spectra were recorded using 1650 UV device spectrophotometer and by dispersing samples in ethanol with 1mg/16 ml concentration at room temperature. The Circular dichroism spectra of the compounds were measured using the J-810 JASCO CD spectrometer at ambient temperature. The surface morphology of the synthesized CTFs were evaluated by scanning electron microscopy (FESEM, Tescan, Czech Republic) equipped with a vacuum at voltage of 10 kV. To record SEM images, samples were sonicated in water (0.1 mg/ml) for 30 seconds. After spreading the solution on the aluminum substrate, they were dried at room temperature and coated with gold. Transmission electron microscopy (TEM) images were recorded using transmission electron microscope (Zeiss EM900). For TEM images water solution (0.1 mg/ml) of samples was sonicated for 30 seconds and dropped on holder. After evaporation of solvent, sample was used for TEM imaging. The crystallinity of CTFs were determined by X-ray diffraction using Cu Kα radiation a Philips X’Pert Pro X-ray diffraction (λ = 1.5418 Å). The thermal behavior of materials were investigated by thermal gravimetric analysis (TGA) model Q600 at temperatures range 25-800 ºC with a 20 ºC/min heating rate under argon atmosphere. The surface area and nitrogen adsorption isotherms were obtained by the Belsorp-mini II device at a temperature of 77 K. Ball milling reactions were performed (300 rpm for 3 hours) by the planetary ball mill (NARYA- MPM 4*250). In order to heat the synthetic compounds up to 100 °C and 400 °C, a VACUUBRAND HP40B2 tube furnace armed with nitrogen inert gas was used.

**Figure. S1.** SEM images a) and IR spectrum b) of the obtained product from reaction of melamine and cyanuric chloride in the absence of Cobalt.


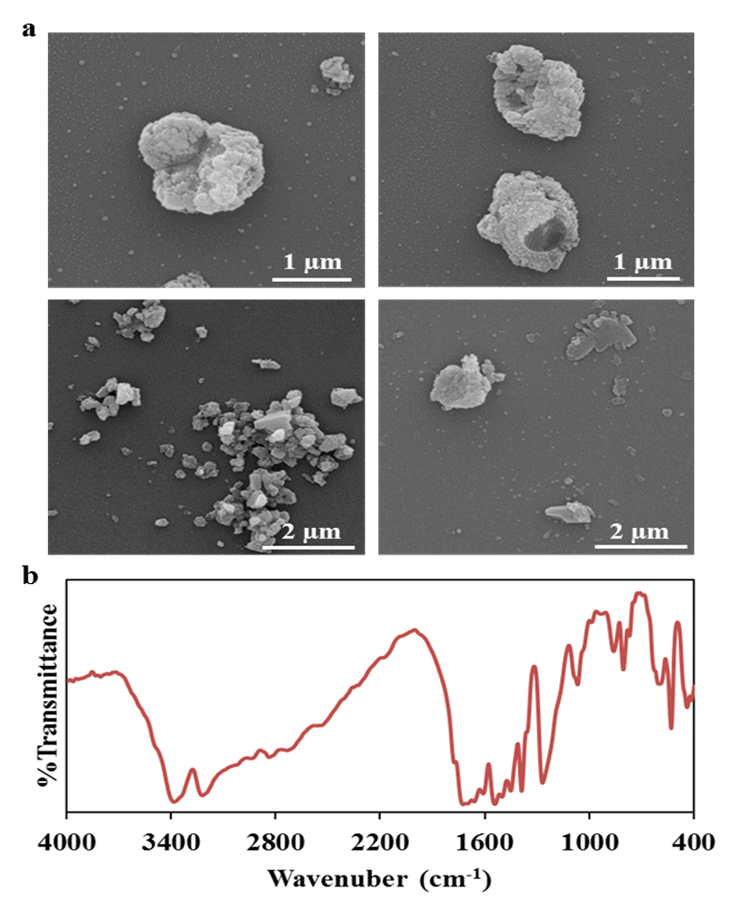

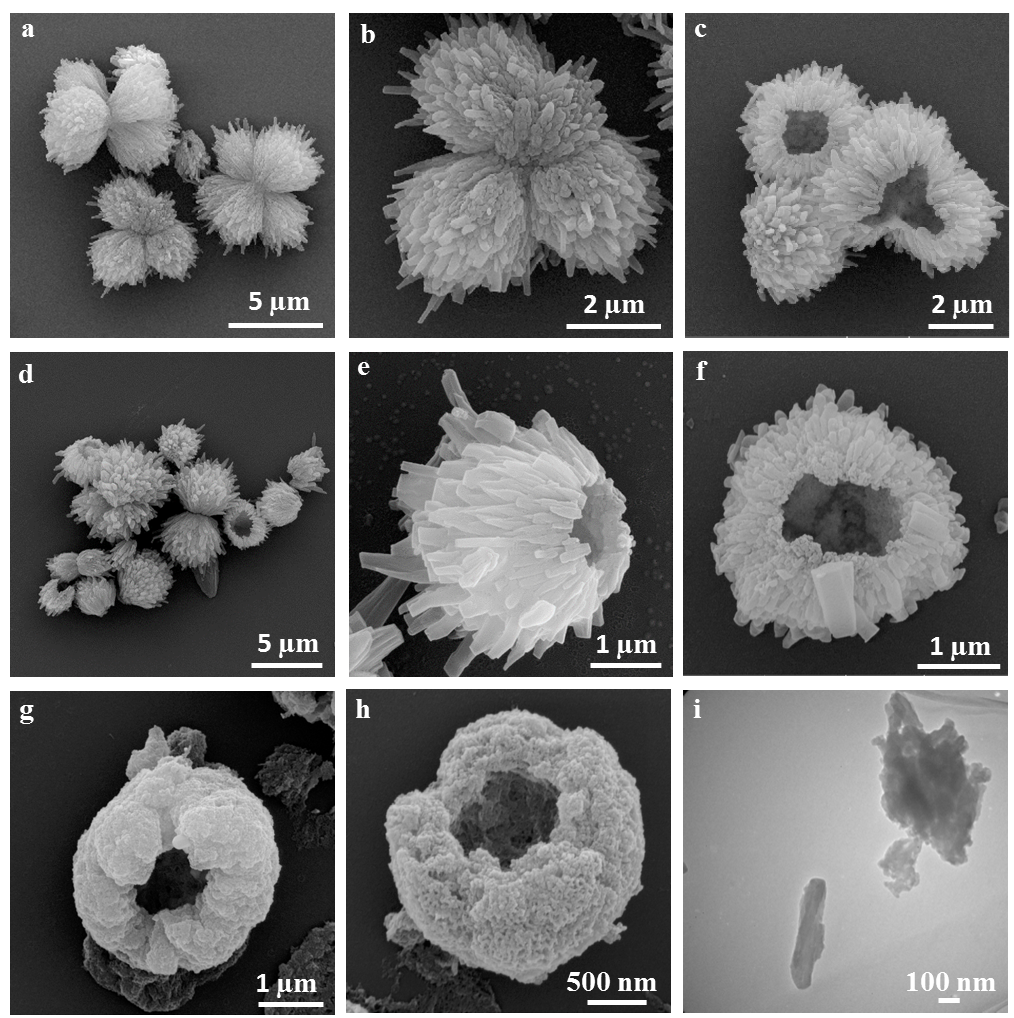


**Figure. S2**. SEM images of triazine covalent organic frameworks synthesized in solution. a, b, c, d) SEM images of PT_Co20_ showing dumbbell-like morphologies. e, f) SEM images of PT_Co100_ displaying calix morphology. g, h) SEM images of PT_Co400_ with calix morphology. i) TEM image of PT_Co100_ demonstrating rod-like building blocks for the 3D structure of this framework.


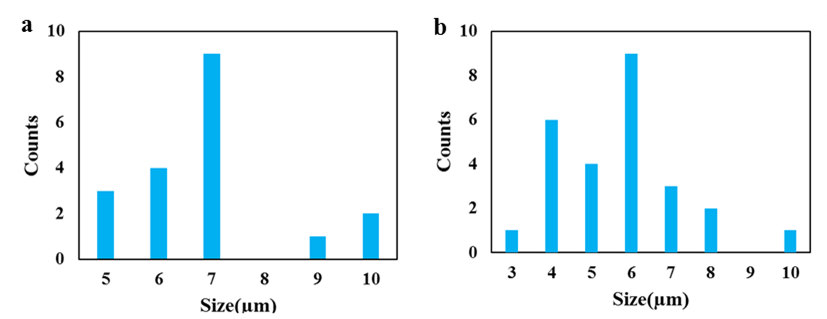


**Figure. S3.** Size distribution of a) PT_Co20_ and b) PT_Co100_ frameworks synthesized in solution.


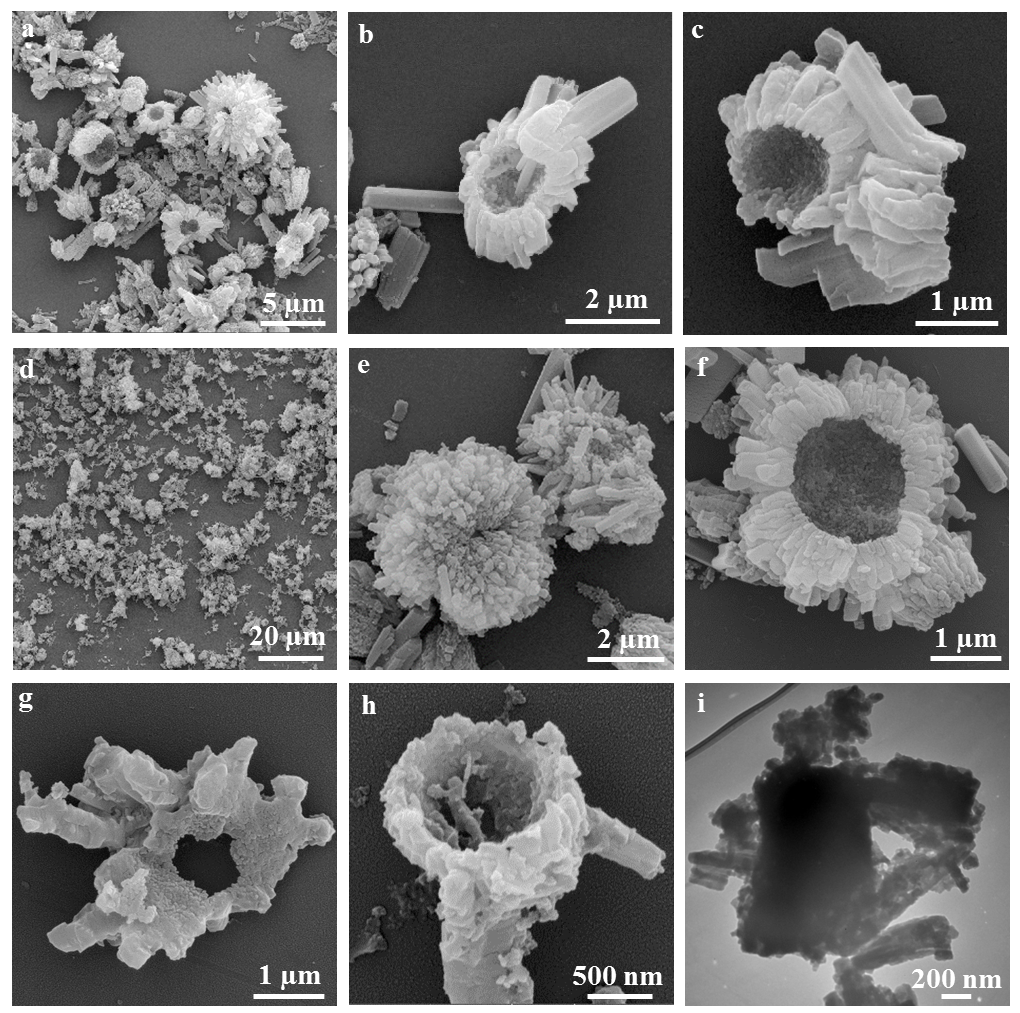


**Figure. S4.** SEM images of mechanochemical synthesized samples. a, b, c) SEM images of MPT_Co20_ show calix morphology. d, e, f) SEM images of MPT_Co100_ showing. g, h) SEM images of MPT_Co400_. i) TEM image of MPT_Co100_ shows a rod-like structure.


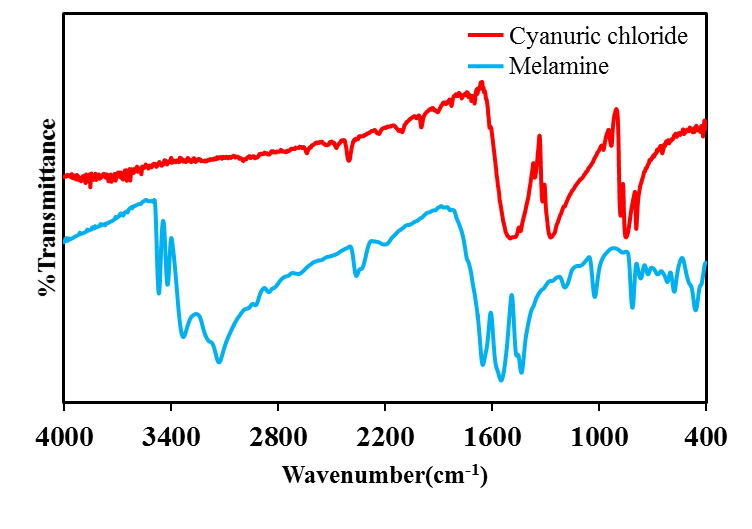


**Figure. S6.** IR spectra of melamine and cyanuric chloride.


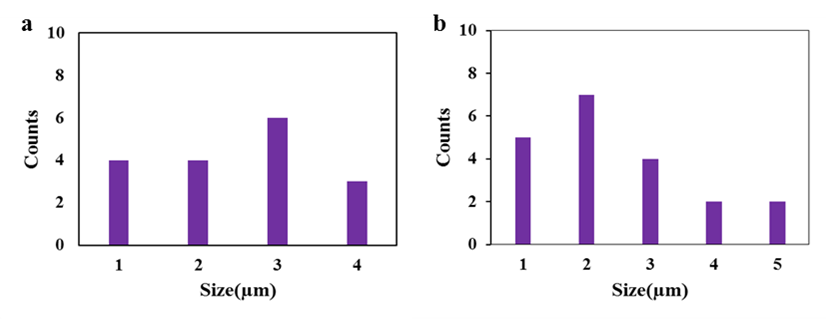


**Figure. S5.** Size distribution of a) MPT_Co20_ and b) MPT_Co100_ frameworks synthesized by mechanochemical method.


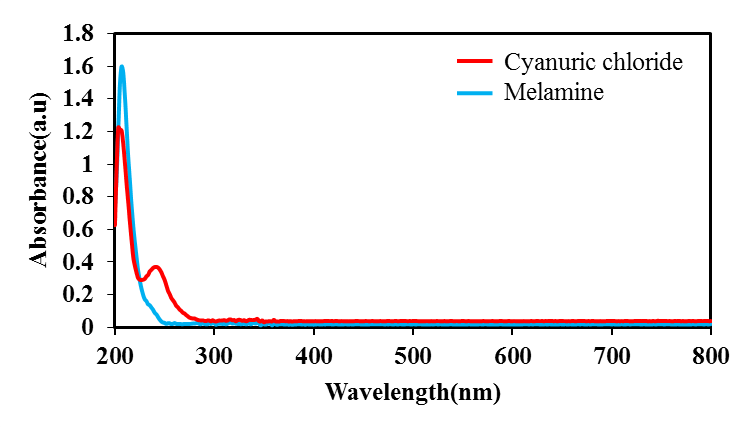


**Figure. S7.** UV-Vis spectra of melamine and cyanuric chloride in ethanol.


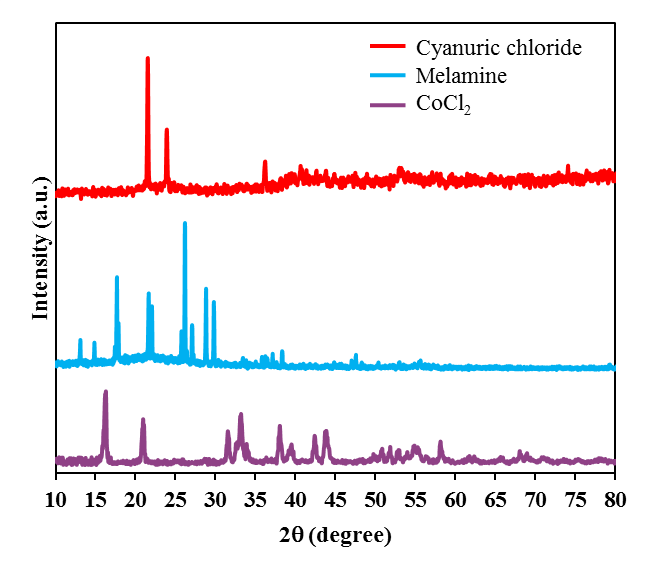


**Figure. S8.** XRD diffractograms of CoCl_2_, melamine and cyanuric chloride.

**Table. S1.** Elemental analysis (EDX) of different compounds synthesized in solution. Carbon to nitrogen ratio (C/N) for PT_Co20_, PT_Co100_, and PT_Co400_ (experimental and calculated) are shown in table.

| Compound | C | N | O | Co | Cl | C/N (Exp.) | C/N (Cal.) |
| --- | --- | --- | --- | --- | --- | --- | --- |
| PT_Co20_ | 28.67 | 54.31 | 16.84 | 0.09 | 0.10 | 0.5 | 0.6 |
| PT_Co100_ | 28.10 | 52.59 | 19.05 | 0.12 | 0.14 | 0.5 | 0.6 |
| PT_Co400_ | 41.85 | 49.46 | 8.47 | 0.16 | 0.06 | 0.8 | 0.6 |

**Table. S2.** Elemental analysis (EDX) of compounds synthesized by mechanochemical method. Carbon to nitrogen ratio (C/N) for MPT_Co20_, MPT_Co100_, and MP_Co400_ (experimental and calculated) are shown in table.

| Compound | C | N | O | Co | Cl | C/N (Exp.) | C/N (Cal.) |
| --- | --- | --- | --- | --- | --- | --- | --- |
| MPT_Co20_ | 31.05 | 51.69 | 17.15 | 0.07 | 0.04 | 0.6 | 0.6 |
| MPT_Co100_ | 30.65 | 52.19 | 17.03 | 0.07 | 0.06 | 0.5 | 0.6 |
| MPT_Co400_ | 32.20 | 53.90 | 13.70 | 0.14 | 0.05 | 0.6 | 0.6 |

**Table. S3.** Elemental analysis (EDX) of the product of control reaction and carbon to nitrogen ratio (C/N) (experimental and calculated) for this compound.

| Compound | C | N | O |  | Cl | C/N (Exp.) | C/N (Cal.) |
| --- | --- | --- | --- | --- | --- | --- | --- |
| Control reaction | 28.69 | 55.32 | 15.89 |  | 0.10 | 0.5 | 0.6 |
|  |  |  |  |  |  |  |  |


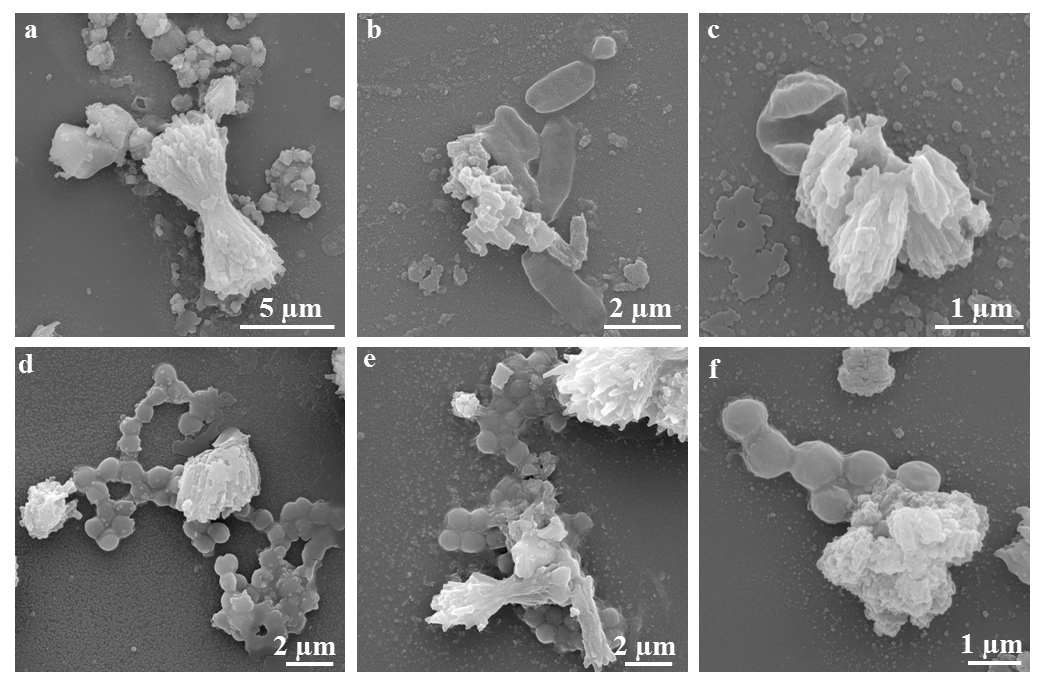


**Figure. S9.**  SEM images of PT_Co100_ and b,c) MPT_Co100_ incubated with E. coli. d,e) SEM images of PT_Co100_ and f) MPT_Co100_ incubated with S. aureus bacteria.

**
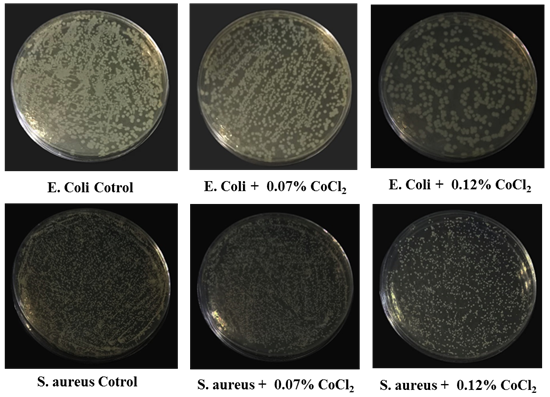
**

**Figure. S10.** Results of antibacterial activity of CoCl_2_ with two amounts of 0.07 and 0.12% w/v incubated with E. coli and S. aureus bacteria.


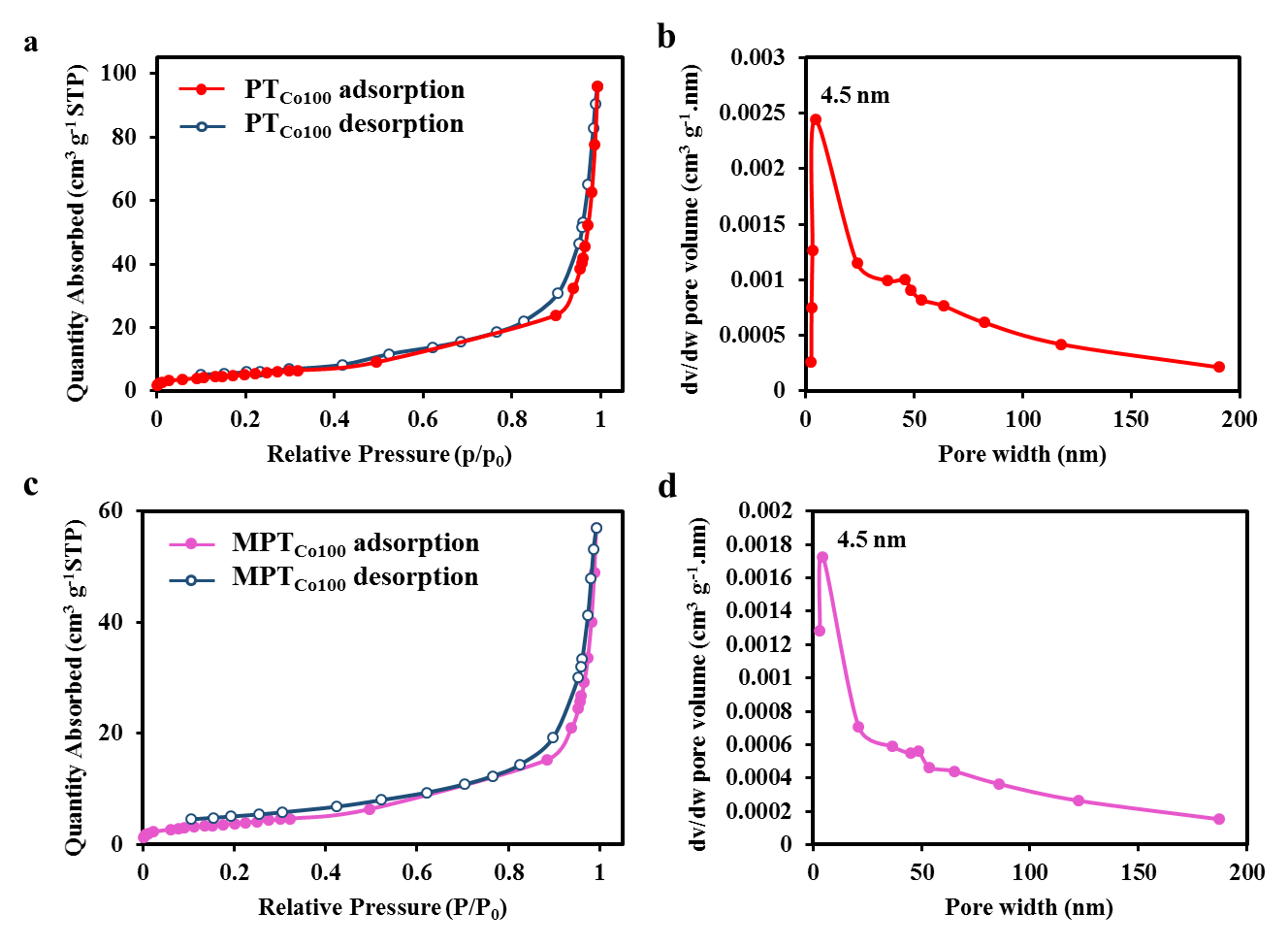


**Figure. S11.** N_2_ adsorption and desorption isotherms of, a) PT_Co100_ and c) MPT_Co100_. Pore ​​size distribution of b) PT_Co100_ and d) MPT_Co100_.
